# Silicone Oil Tamponade Removal: Which Technique Is More Effective? An X-Ray Photoemission Spectroscopy Study

**DOI:** 10.1167/tvst.12.4.21

**Published:** 2023-04-18

**Authors:** Tommaso Rossi, Paolo Canepa, Ornella Cavalleri, Ranieri Rolandi, Giorgio Querzoli, Isabella D'Agostino, Serena Telani, Guido Ripandelli

**Affiliations:** 1 IRCCS Fondazione Bietti–ONLUS, Rome, Italy; 2 Department of Physics, University of Genoa, Genoa, Italy; 3 DICAAR Università di Cagliari, Cagliari, Italy; 4 ASL 3 Liguria–Ospedale Antero Micone, Genova, Italy

**Keywords:** pars plana vitrectomy, silicone oil tamponade, silicone oil removal, X-ray photoemission spectroscopy, retinal detachment

## Abstract

**Purpose:**

To compare the efficacy of two surgical techniques used to remove silicone oil (SiO) emulsion tamponade after pars plana vitrectomy: triple air–fluid exchange (AFX) and balanced salt solution lavage (BSSL).

**Methods:**

X-ray photoemission spectroscopy measured silicon content of the dry residue of fluid samples taken during AFX and BSSL. Ten patients underwent AFX and five BSSL. Three fluid samples were taken per patient, and the dry residue of 10 drops per sample were analyzed. A fluid sample from a patient who never received SiO tamponade was also analyzed to set a “blank” reference sample.

**Results:**

Patients’ demographics showed no significant difference. Sample 1 of the two groups contained comparable silicon content while samples 2 and 3 of the AFX group contained significantly more silicon than that of the BSSL group (15.0 ± 0.1 and 12.0 ± 0.9 for the AFX group vs. 10.7 ± 1.4 and 5.2 ± 0.6 for the BSSL group, respectively; *P* < 0.05). The cumulative amount of silicon in the three successive samples was also significantly higher for the AFX group (42.3 ± 1.6 vs. 32 ± 2; *P* < 0.0001). The average silicon content ratio of consecutive samples was significantly higher for the AFX group compared to the BSSL group (0.90 ± 0.01 vs. 0.58 ± 0.06; *P* = 0.006).

**Conclusions:**

Triple AFX removed more silicon than triple lavage. The eye wall actively interacts with silicon emulsion retaining silicon content rather than behaving as a neutral container.

**Translational Relevance:**

Triple air–fluid exchange removed more silicon than BSS lavage. Neither technique behaved as a well-mixed box dilution, suggesting the eye walls actively retain emulsion and a dynamic equilibrium is established between silicon dispersion and the eye wall surface.

## Introduction

Polydimethylsiloxane (PDMS or silicone oil, SiO) is used as a long-term vitreous substitute after pars plana vitrectomy (PPV) for a variety of surgical indications, including retinal detachment and diabetic and proliferative vitreo-retinopathy.^1^

Tamponade duration may vary according to clinical presentation,^2^ but usually SiO–fluid exchange is performed within 3 to 6 months.^3^ Inflammation mediators generated by disease and surgery itself emulsify SiO into droplets^4^ whose removal is important to minimize trabecular meshwork damage, visual symptoms, and long-term complications, including glaucoma, corneal decompensation, and SiO migration to the central nervous system.^5^

Silicone oil removal can be achieved through different surgical techniques,^6^ all intended to remove as much emulsion as possible^7^: the isovolumetric SiO–fluid exchange is followed by multiple consecutive air–fluid exchange (AFX) or balanced salt solution lavage^8^ (BSSL). Despite a lengthy use of SiO as a vitreous substitute, it is still unclear which is the most effective removal technique.^9^

X-ray photoemission spectroscopy (XPS, described in Appendix A) is a highly sensitive technique^10^ able to identify elements present in a sample at concentrations down to a few tenths of a percentage.^11^ It measures the kinetic energy of the photoelectrons emitted by the sample upon interaction with the X-ray beam. From the kinetic energy, the binding energy of the photoelectron can be calculated, which uniquely identifies the chemical element that emitted the photoelectron. XPS has been largely and successfully used for the analysis of inorganic^12^ and bio/organic compounds and interfaces.^13^^,^^14^

The purpose of this article is to compare the efficacy of triple AFX and BSSL in removing silicon oil emulsion from the vitreous chamber by performing XPS in three consecutive intraocular fluid samples, withdrawn during the two different techniques.

## Materials and Methods

### Surgical Technique and Sample Collection

Fifteen consecutive patients undergoing SiO removal after PPV for retinal detachment with proliferative vitreo-retinopathy grade C were randomized to undergo either a triple air–fluid exchange (AFX group) or vitreous chamber lavage with balanced salt solution (BSS; Beaver Visitec Medical, Waltham MA, USA) (BSSL group) in a 2:1 ratio. All patients previously received 1000 centistokes (cSt) SiO tamponade (PDMS Micromed, Rome, Italy). Myopic eyes (more myopic than 3 diopters) were excluded to avoid significant differences in vitreous chamber volume.

In both AFX and BSSL techniques, the macroscopic SiO bubble was thoroughly aspirated with the phaco-vitrectomy machine Optikon R-Evolution 800CS (BVI; Beaver Visitec Medical) using 23-gauge active aspiration. After no visible macroscopic SiO bubble was floating within the vitreous chamber, the two groups differed in the following:

(1) AFX group underwent triple AFX—that is, air infusion was turned on and the entire fluid present within the vitreous chamber was exchanged to air and collected into a sterile vial immediately sealed and labeled. Then, the BSS infusion was turned on and air was allowed to escape from the valved trocar until the vitreous chamber was completely filled with the BSS inflowing from the infusion line. The intraocular fluid volume removed during each of the three consecutive fluid–air exchanges (around 4 mL) was withdrawn with a reusable sterile glass syringe collected in sterile containers and labeled for the successive analysis.
(2) The BSSL group underwent BSS lavage of the vitreous chamber performed as follows: 4 mL BSS was withdrawn from the vitreous chamber through valved 23-gauge trocars into a syringe, leaving the BSS infusion open, in order to immediately restore volume, therefore diluting the existing microscopic emulsion and paying attention to avoid spill and leakage to minimize unintended dilution. The same procedure was repeated three times to collect three successive BSS samples of 4 mL volume each within a reusable sterile glass syringe, immediately stored into sealed vials, and labeled as samples 1 to 3.

Both air and BSS infusion pressure were set at 30 mm Hg throughout the procedures.

The purpose of a triple 4-mL lavage was to match the triple AFX group in terms of BSS volume exchange, assuming a 4-mL typical vitreous chamber volume. Valved trocars were used in all cases to minimize fluid leakages during the procedure.

We also examined the silicon content of a BSS lavage fluid sample of a patient undergoing macular surgery who never received SiO tamponade (blank sample) to confirm the significance of XPS measures in detecting the presence of SiO. The BSS directly deposited on aluminum foil and the BSS deposited after passing through a syringe showed a silicon content compatible with the silicon content of the blank sample. The study followed the tenets of the Declaration of Helsinki and received institutional review board approval; all patients signed an informed consent for both the surgical procedure and intraocular fluid examination, and the study was registered at www.clinicaltrials.gov (NCT04774146).

### Sample Analysis

Fluid specimens were immediately sealed into vials to prevent evaporation, sent to the laboratory, and examined within 48 hours. Each vial was shaken for 1 minute before depositing 10 drops onto an aluminum foil. After deposition, samples were dried overnight in a desiccator in order for the watery solvent to evaporate, leaving any nonvolatile material deposited over the aluminum foil. Each droplet had a volume of 0.3 µL, with a resulting diameter of the dried droplet of about 400 µm. The chemical composition of the dry residue of each droplet was investigated by XPS analysis. XPS measurements were carried out using a PHI 5600 Multi-Technique apparatus (Physical Electronics, Inc., Eden Prairie, MN, USA), equipped with an X-ray Al-monochromatized source (hν = 1486.6 eV). The footprint of the X-ray beam was about 400 µm in diameter, comparable to the diameter of each dried droplet. Spectra of the Si2p, C1s, Na1s, and Cl2p core level regions were acquired using a pass energy of 58.7 eV. A neutralizer (low-energy electron flood gun) was used during measurements to avoid sample charging.^15^ Spectra analysis was performed with the PHI proprietary MultiPak Spectrum software (Physical Electronics, Inc.). The binding energy scale was calibrated by setting the C1s component of adventitious carbon at a binding energy of 284.8 eV. The amount of each element was measured as the intensity of the XPS signal related to each element weighted by the specific element sensitivity.

We measured the SiO content in the samples derived from consecutive AFX or BSSL as the percentage of silicon (Si) with respect to the total amount of Si, carbon (C), sodium (Na), and chlorine (Cl): Si/(Si + C + Na + Cl) × 100. Na and Cl are representative of the BBS used for fluid–air exchanges and lavages.

### Mathematical Model

The two mathematical models are discussed in detail in Appendix B; they rely on the so-called well-mixed box assumption, where SiO is supposed to be uniformly distributed in the fluid within the eye and any discrete volume of fluid removed, and the container (eye wall) is supposed to not interact with the fluid content.

When performing AFX, the surgeon almost empties the vitreous chamber, removing a fraction α of the fluid with its content of SiO mass, so that only a small amount remains; then the chamber is replenished with clear BSS that will dilute the remaining SiO mass. After *n* AFXs, the mass of SiO still present in the eye will be

$$\begin{array}{c} m_{n}=m_{0}{\left(1-\alpha \right)}^{n} \end{array}$$

where *m*_0_ is the amount of SiO at time 0. We are interested in quantifying the amount of SiO mass removed with the surgical technique during each exchange (Δ*m_n_*). Thus, using the above equation, we express the ratio of the content of SiO in two consecutive samples as follows (see Appendix B):

$$\begin{array}{c} \frac{\Delta m_{n}}{\Delta m_{n-1}}=\left(1-\alpha \right) \end{array}$$

BSS lavage, on the other hand, can be considered the continuous replacement of minuscule SiO emulsion volumes containing minuscule SiO mass with clear BSS. The infinitesimal amount of SiO (*dm*) removed with the infinitesimal volume *dV* equals the concentration times the volume *dV*. Thus, replacing a volume of fluid *dV* causes a decrease of *dm* in the mass of SiO remaining in the chamber:

$$\begin{array}{c} dm=-CdV=-\frac{m}{V_{0}}dV \end{array}$$

Again, we are interested in amount of the mass (*Δm_n_*) of SiO removed during a lavage with a volume *V*_0_ of fluid, which represents our sample data. Thus, using the solution of the differential equation reported in Appendix B, we express the ratio of mass removed in two consecutive lavages (namely, the *n*th and (*n* – 1)th) as

$$\begin{array}{c} \frac{\Delta m_{n}}{\Delta m_{n-1}}=\frac{1}{e} \end{array}$$

As a result, if the vitreous chamber behaved as a well-mixed box, the ratio between successive samples would be a constant equal to (1 – α) in the case of AFX and equal to 1/*e* in the case of BSSL.

### Statistical Analysis

The results are expressed as mean ± standard error. Statistical analysis used analysis of variance and *t*-test for repeated measures with Bonferroni correction for multiple comparisons. In all cases, *P* values less than 0.05 were considered statistically significant.

## Results

The demographics of the AFX and lavage groups are reported in Table 1: no statistically significant difference was found for age, sex, tamponade duration, surgery duration, risk of re-detachment, and final visual outcome. Silicon content of the blank samples was 3.9% ± 1.5%.

**Table 1.** Demographics and Surgical Data

Characteristic	BSS Lavage Group	Air–Fluid Exchange Group	*P* Value
Number of patients	5	10	—
Age, mean ± SE	57 ± 14	61 ± 11	ns
Males/females, *n*	2/3	6/5	ns
Silicone oil tamponade duration, mean ± SE, d	148 ± 42	139 ± 59	ns
Final visual acuity (Snellen/LogMAR)	20/58 0.46	20/62 0.49	ns
SiO removal surgery duration, mean ± SE, min	29 ± 12	31 ± 9	ns
Retinal detachment after SiO removal, *n*	0/5	1/10	ns

**Figure 1.**
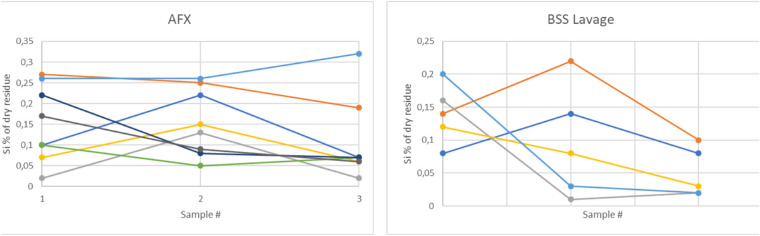
Average silicon content of all samples divided by group. Different colors indicate different patients. The *red line* indicates the blank reference.

The silicon content of fluid samples belonging to the patients of both groups is reported in Figure 1; all samples belonging to both groups contained more silicon than the blank sample.

**Figure 2.**
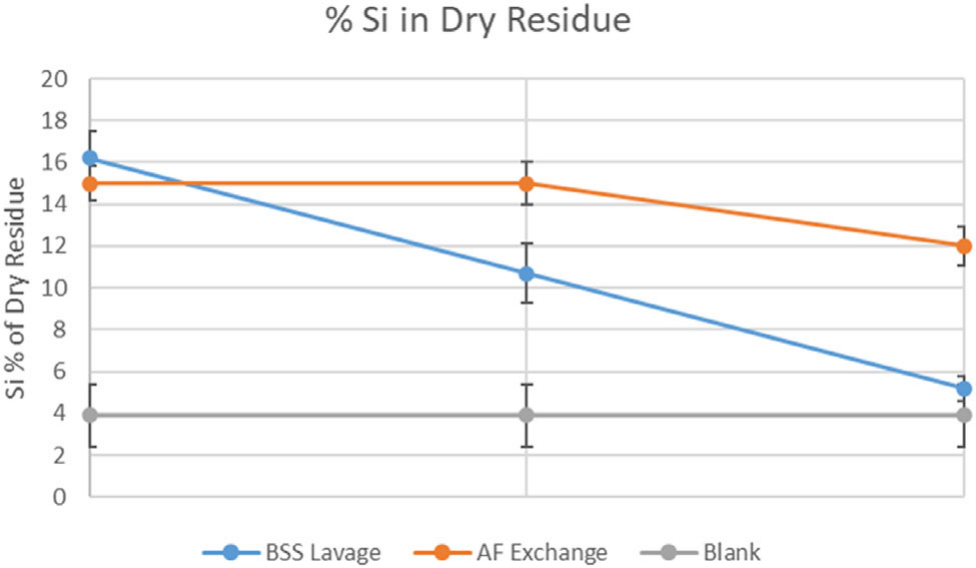
Silicon content per sample and across groups. The difference in silicon content of samples 2 and 3 is significant.

Figure 2 and Table 2 report the sample mean by group: average silicon content of sample 1 did not differ significantly across groups, while samples 2 and 3 of the AFX group contained significantly more silicon than the BSSL group. Average sample silicon content across consecutive samples of both groups decreased significantly between samples 1 and 3 (*P* = 0.011 for AFX and *P* < 0.001 for BSSL).

**Table 2.** Comparison of Average Silicon Content of Consecutive Samples

Characteristic	BSSL Group, Mean ± SE	AFX Group, Mean ± SE	*P* Value
Silicon content sample 1	16.2 ± 1.3	15.0 ± 0.8	0.434
Silicon content sample 2	10.7 ± 1.4	15 ± 1	0.013
Silicon content sample 3	5.2 ± 0.6	12.0 ± 0.9	< 0.0001

**Figure 3.**
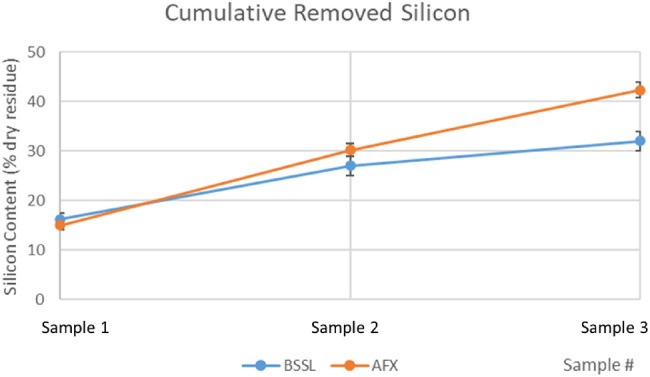
Cumulative silicon content per group. The difference of samples 1 + 2 + 3 is significant.

The mean cumulative amount of silicon (samples 1 + 2 + 3) removed by means of AFX was significantly higher than BSS lavage (Fig. 3 and Table 3).

**Table 3.** Cumulative Removed Silicon Mass

Characteristic	BSSL Group, Mean ± SE	AFX Group, Mean ± SE	*P* Value
Sample 1	16.2 ± 1.3	15.0 ± 0.8	0.434
Sample 1 + 2	27 ± 2	30.2 ± 1.3	0.071
Sample 1 + 2 + 3	32 ± 2	42.3 ± 1.6	<0.0001

The average silicon content ratio between consecutive samples was significantly higher for the AFX group compared to the BSSL group (0.9 ± 0.01 vs. 0.58 ± 0.06; *P* = 0.006; Table 4).

**Table 4.** Silicon Mass Ratio of Consecutive Samples and Average as a Function of Surgical Technique

Characteristic	BSSL Group, Mean ± SE	AFX Group, Mean ± SE	*P* Value
Sample 2/sample 1	0.7 ± 0.1	1.017 ± 0.08	0.016
Sample 3/sample 2	0.49 ± 0.09	0.79 ± 0.08	0.013
Average	0.58 ± 0.06	0.9 ± 0.1	0.006

AFX ratios are larger than BSSL ratios.

## Discussion

The surgical techniques used to remove SiO emulsion^7^ include triple air–fluid exchange and BSS lavage,^8^ intended to dilute progressively the SiO emulsion. To compare the two, we standardized BSS lavage to a “triple” withdrawal of a volume equal to the volume of the typical vitreous chamber (V_0_ = 4 mL).

Previous studies comparing SiO removal techniques were based on the qualitative measure of patients’ floaters,^5^ emulsion droplets,^4^ or scattering particles in ultrasound video frames,^16^ but none quantitatively measured silicon content. Examining the dry residue through XPS allowed unprecedented accuracy, although it also identified the small “background” silicon quota physiologically present in human fluids^17^; to discriminate the two fractions, we measured samples of a patient who never received SiO tamponade as a blank reference.

Sample 1 experimental data showed a wide distribution of silicon content across patients of both groups (Fig. 1), representative of the largely variable “baseline” emulsion at the time of surgery^18^ but no difference between groups, as expected. Samples 2 and 3 of the AFX group showed a significantly higher silicon content (Fig. 2 and Table 2), and AFX technique allowed the removal of a significantly higher cumulative silicon mass (Fig. 3 and Table 3).

**Figure 4.**
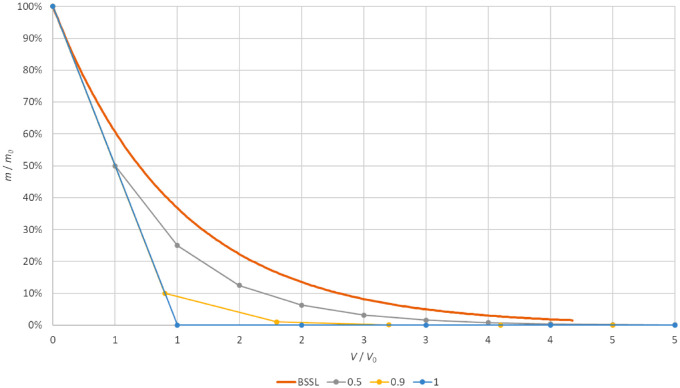
Theoretical silicon oil mass decrease (y-axes) in a well-mixed box model as a function of the total exchanged volume, *V*, normalized by the vitreous chamber volume, *V*_0_ (x-axes), for BSSL (*orange curve*) and AFX assuming three different levels of volume fraction removal (*a* is the fraction of the total vitreous chamber volume removed during each air–fluid exchange): complete ocular volume removal (α = 1, *blue curve*) and 90% or 50% removal (*a* = 0.90 and *a* = 0.50, *yellow* and *gray* *curves*, respectively; see Appendix B for details).

Assuming that SiO is removed in the same proportion as fluid (i.e., in the scenario where oil droplets are randomly dispersed in the BSS and the surgical probe collects randomly BSS and oil without any “preference”), both BSSL and AFX should follow the dilution laws of a well-mixed box (Fig. 4): BSS lavage is a continuous replacement of mixture with clear fluid, whereas AFX consists of two asynchronous phases, a nearly complete removal of the mixture followed by the injection of an equivalent volume of clear fluid. The main difference between BSSL and AFX is the way the solvent is introduced.

Silicon content ratio of consecutive samples is a sensitive indicator of the procedure efficacy, and its value is expected to be constant among consecutive samples in the well-mixed box scenario and can be calculated equal to 1/*e* for BSS lavage and (1 – α) for the AFX well-mixed models, respectively (see Appendix B).

**Figure 5.**
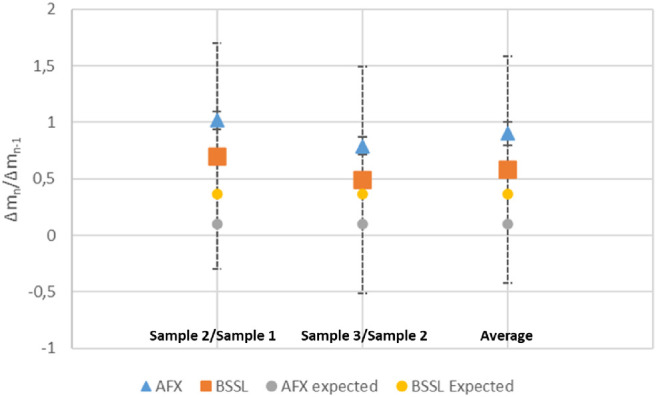
Average ratio of successive samples in the case of AFX and BSSL compared to the expected theoretical values.

Silicon content ratio of experimental data reported in Table 4 and Figure 5 shows that both groups performed much worse than their respective models: if the eye behaved as a neutral container and followed the well-mixed box model, in fact, more effective procedures would give lower successive sample ratios.

The rapid decrease of the SiO content, given by the α = 0.90 procedure (Fig. 4), corresponds to a ratio of the different samples as small as 0.1, much lower than the ratio (0.5) resulting from the α = 0.50 procedure; in the same scenario, BSSL, which in Figure 4 exhibits the slower decrease of SiO content, would give the lower ratio: 0.37 (corresponding to 1/*e*). Real-life emulsion behaves much differently since our experimental data show an average silicon ratio between consecutive samples of 0.58 for BSS lavage and 0.9 for AFX (Table 4, Fig. 5), both much higher than expected in a well-mixed box, meaning that both assumptions of silicon removal in the same proportion as fluid and the eye wall as a neutral container are false and must be rejected.

There are two main reasons explaining this macroscopic deviation from the well-mixed box model: the nonneutral role of the eye wall in retaining SiO and the active search by the surgeon of SiO emulsion floating over the fluid–air interface during AFX. In principle, the surgeon actively seeking SiO emulsion should have a higher mass removal compared to the box model, whereas the emulsion adherence to the eyewall should decrease the effectiveness of the procedure. Figure 5, comparing expected and observed data, clearly suggests that the second effect dominates and the eye wall “actively retains” silicon: the vitreous chamber is not a “neutral” container.

Therefore, our data indicate that the silicon content of all examined samples represents a fraction of the total intraocular silicon mass present in the eye, and the “eye wall” is far from being neutral but behaves as a “buffer” dynamically exchanging SiO emulsion with its liquid content according to complex (and still unknown) mechanisms.

It should be noted that the macroscopic SiO bubble and its emulsion contact several anatomic structures, including the optic nerve, retina, pars plana, pars plicata, ciliary body, posterior iris, Zinn's zonule, and crystalline lens posterior capsule, whose intricate anatomy, roughness, polarity, and charge distribution can hide, enclose, or bind SiO emulsion.

The relative efficiency of both methods compared to their respective theoretical model is shown by the distance between experimental data and the model in Figure 5. Why so little silicon is removed during the triple AFX compared to its theoretical model (Fig. 4 and Fig. 5) remains puzzling: emulsified SiO adheres to biologic tissues^1^; when fluid is exchanged to air, the increase in surface tension may favor droplet adherence to rougher surfaces such as the pars plicata and iris. Similarly, the supernatant SiO at the fluid–air interface may “seed” the emulsion over the eye walls as fluid is removed just like seashells left on the shore. This may explain the mechanism of the SiO “buffer” and the presence of silicon in comparable concentrations throughout the subsequent samples discussed in detail in Appendix B. It should also be noted that the roughness of the inner vitreous chamber, especially at the iris and pars plicata, makes its surface incomparably wider than a sphere portion.

Shiihara et al.^16^ concluded that AFX decreased its effectiveness compared to BSSL as the axial length increased: a notion consistent with the above argument since longer axial length determines a wider surface. Yu et al.^8^ compared the number and size of droplets after AFX and BSSL using a Coulter counter and reported a 35% to 40% of reduction in SiO bubbles between 1 and 12 microns. This figure does match our results. However, it should be noticed that Yu et al.^8^ considered only a range of droplet sizes, whereas XPS allows a much more accurate detection of SiO.^4^ As a matter of fact, the total SiO content is related not only to the droplet size but also to their number. Therefore, it is not possible to assess the SiO contained in the droplets not considered in their count.

It is worth noting that we do not know the overall silicon content at time 0 (*m*_0_), nor we can reliably estimate it. However, the ratio of successive samples (Δ*m*_3_/Δ*m*_2_ and Δ*m*_2_/Δ*m*_1_) that we measure is quite high, compatible with a very low overall silicon removal efficacy (Δ*m*/*m*_0_; i.e., the ratio of the diminution of silicon mass to the initial mass) of both techniques, presumably less than 10% to 20%. This notion suggests that regardless of the technique used, the surgeon should probably aim at introducing a solvent volume at least five to six times the vitreous chamber.

**Figure 6.**
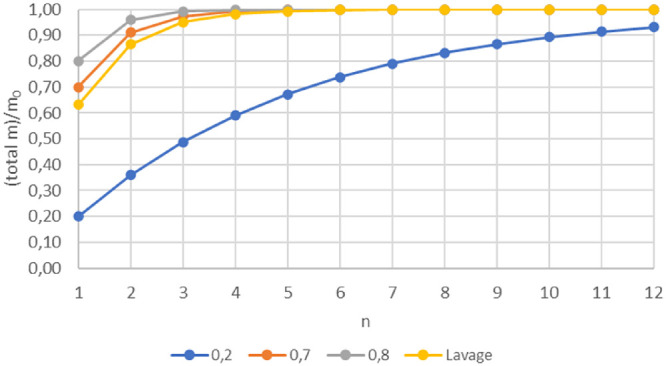
Comparison of silicon cumulative removal of BSS lavage (*yellow curve*) compared to air–fluid exchange hypothesizing silicon removal efficacy of 0.2 (*blue*), 0.7 (*orange*), and 0.8 (*gray*). *n* is the number of exchanges for AFX and the ratio of the fluid used during the lavage to the chamber volume in the case of BSSL. Note that with very low AFX efficacy (0.2), it would take 12 exchanges to approach the lavage capability of cumulative silicon removal.

Although AFX removed more silicon than lavage, it proved less efficient than BSSL when compared to its respective theoretical model. This may be clinically relevant since it is probably easier and less surgically dangerous to prolong BSS lavage than to perform multiple air–fluid exchanges in order to increase the fraction of removed SiO (Fig. 6).

It is also conceivable that the infusion cannula laminar “jet flow” impinging the retina during BSS lavage may favor the detachment of SiO emulsion adherent to it and its successive aspiration, while the air bubble generated during the air exchange may result in laying a uniform coating of SiO over the vitreous chamber walls. If this is proved correct, a directional cannula held by the surgeon and “sweeping” the vitreous chamber walls may increase the effectiveness of the procedure.

In summary, for the first time, we used XPS as an objective and quantitative measure of silicon content in the subsequent intraocular fluid samples collected during SiO emulsion removal with two different surgical techniques. AFX removed a higher mass of silicon across the three lavages compared to BSS lavage, but none behaved as expected unless we postulate that the eye walls have a high “intrinsic” capability of interaction and retainment of SiO during the procedure.

Our data suggest that SiO dispersion establishes a complex relationship with the eye wall, with biologic, chemical, and electrostatic properties playing an important role in the removal dynamics.

The pitfalls of present study include the relatively small sample size and the novelty of XPS for the present purposes that find very few, if any, comparable studies in the literature. Also, it should be noted that 1000 cS SiO was used for all our patients, and therefore conclusions may not apply to different viscosity compounds. On the other hand, XPS is a sensitive method extensively used for other purposes, and each measure is based on readings from 10 droplets of the same sample to increase data robustness.

## Figures and Tables

**Figure A1.**
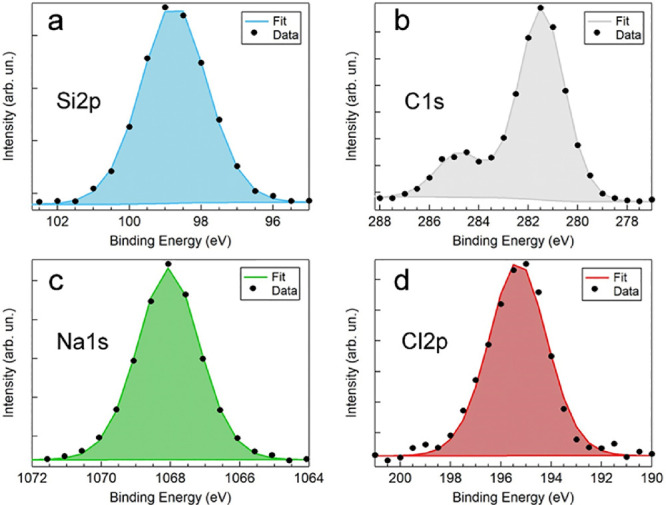
Typical XPS spectra of the Si2p, C1s, Na1s, and Cl2p core level regions of dried intraocular fluid samples deposited on an Al foil.

## Appendix A Appendix A

XPS is a highly sensitive technique able to identify elements present in a sample at concentrations down to a few tenths of a percentage. It measures the kinetic energy of the photoelectrons emitted by the sample upon interaction with the X-ray beam. From the kinetic energy, the binding energy of the photoelectron can be calculated, which uniquely identifies the chemical element that emitted the photoelectron. XPS has been largely and successfully used for the analysis of inorganic and bio-organic materials. It operates in ultra-high-vacuum conditions and exploits the photoelectric effect (i.e., the emission of core electrons from the atoms of a sample when it is irradiated with an X-ray beam). By measuring the kinetic energy of the emitted photoelectron (KE), the binding energy (BE) can be obtained through the following equation:

$$\begin{array}{c} \mathrm{BE}=h\nu -\mathrm{KE}-\Phi \end{array}$$

where h*ν* is the photon energy and Φ is the spectrometer work function. A typical XPS spectrum reports the number of photoelectrons as a function of their BE. Each element produces a set of characteristic XPS peaks, which correspond to the energy level of the electrons within the atom and are influenced the chemical environment of the atom. The analysis of XPS spectra enables identification and quantification of all surface elements (except hydrogen and helium), with a sensitivity down to ∼0.1 at %. To evaluate atomic percentage values, each raw XPS signal is corrected by dividing the signal intensity by a relative sensitivity factor (RSF) and normalized over all elements of interest.

Figure A1 shows typical Si, C, Na, and Cl signals measured on the dried droplets of intraocular fluid samples.

## Appendix B Appendix B

As a support to the interpretation of the present results, we briefly describe two mathematical models based on the assumption that SiO is removed from the vitreous chamber in the same proportion as the fluid: the so-called well-mixed box model, which is well known in the case of a chemical solution. This behavior corresponds to the ideal case of oil droplets uniformly dispersed in the fluid within the vitreous chamber that do not interact with the chamber walls and the probe aspirating BSS and oil with no preference.

First, we consider the case of AFX:

Assume the surgeon performs a series of exchanges, (1) aspirating each time a volume *V_r_* = α × *V*_0_ corresponding to a fraction α of the volume (*V*_0_) of the fluid contained in the chamber and, at the same time, removing the fraction, α, of the SiO present in the chamber and (2) refilling the chamber with BSS to the initial volume of the vitreous chamber *V*_0_.

After the first exchange, we have that the mass of SiO contained in the chamber equals

$$\begin{array}{c} m_{1}=m_{0}\left(1-\alpha \right) \end{array}$$

where *m*_0_ is the initial mass of SiO. At the following exchanges, the same arguments yield

$$\begin{array}{c} m_{2}=m_{1}\left(1-\alpha \right)=m_{0}{\left(1-\alpha \right)}^{2} \end{array}$$

$$\begin{array}{c} m_{3}=m_{2}\left(1-\alpha \right)=m_{0}{\left(1-\alpha \right)}^{3} \end{array}$$

and so on.

Therefore, after the *n*th exchange, the oil mass still present in the eye is

$$\begin{array}{c} m_{n}=m_{0}{\left(1-\alpha \right)}^{n}. \end{array}$$

This equation expresses the mass of SiO remaining in the eye as a function of the number, *n*, of air–fluid exchanges. However, when performing the continuous BSSL, the number of maneuvers is not a significant parameter. As a consequence, we plotted in Figure 4 the oil mass, *m*, as a function of the volume of fluid used in the following exchange: V = *n* × (α × *V*_0_), normalized by the chamber volume, *V*_0_, that is, *V*/*V*_0_ = *n* × α. However, it is virtually impossible to measure the quantity of oil actually present in the eye at the beginning of the procedure. Therefore, we focused on the oil mass removed during the *n*th exchange:

$$\begin{array}{c} \Delta m_{n}=m_{n-1}-m_{n}=m_{0}\left({\left(1-\alpha \right)}^{n-1}-{\left(1-\alpha \right)}^{n}\right) \end{array}$$

because Δ*m_n_* is the mass of SiO that we would find in the *n*th sample in the present study in the ideal case that the above assumptions hold (i.e., no interaction of SiO with the eye walls).

Then, in order to remove the dependence on the initial mass, *m*_0_, which is unknown in our measurement, we obtained from the above equation the ratio of the Si content in successive samples (Fig. 5):

$$\begin{array}{c} \frac{\Delta m_{n}}{\Delta m_{n-1}}=\left(1-\alpha \right) \end{array}$$

A similar argument can be developed for BSSL.

The difference is that, in this case, we consider the lavage as a succession of infinitesimal replacements of oil-contaminated fluid with clear fluid. Assuming that *C* = *m/V*_0_ is the mass of SiO per unit BSS volume at a generic instant, when we aspirate an infinitesimal volume *dV* from the chamber, we remove a proportional mass, *dm*, of SiO. Therefore, we have

$$\begin{array}{c} dm=-CdV=-\frac{m}{V_{0}}dV \end{array}$$

which, dividing by *dV*, leads to the differential equation:

$$\begin{array}{c} \frac{dm}{dV}+\frac{m}{V_{0}}=0 \end{array}$$

Imposing that the initial mass of SiO contained in the chamber is *m*_0_, the above equation has the following solution:

$$\begin{array}{c} m\left(V\right)=m_{0}e^{-\frac{V}{V_{0}}} \end{array}$$

where, as above, *V* indicates the volume of BSS presently exchanged. In the present procedure, we sampled when *V* was an integer multiple *n* = 1, 2, 3 of the chamber volume (i.e., when *V* = *nV*_0_). Consequently, we can rewrite the above equation as

$$\begin{array}{c} m_{n}=m\left(nV_{0}\right)=m_{0}e^{-n} \end{array}$$

and, similarly as above, we can estimate the amount of SiO contained in the *n*th sample:

$$\begin{array}{c} \Delta m_{n}=m_{n-1}-m_{n}=m_{0}\left(e^{-\left(n-1\right)}-e^{-n}\right) \end{array}$$

that is, the quantity that we actually measured in the present study. We can use the above equation to compute the ratio of the Si content in successive samples, thus removing the dependency on the unknown, *m*_0_:

$$\begin{array}{c} \frac{\Delta m_{n}}{\Delta m_{n-1}}=\frac{1}{e}. \end{array}$$

In conclusion, if the vitreous chamber behaved as a well-mixed box (i.e., in the absence of any role of the eye walls), the ratio between successive samples would be a constant equal to 1/*e* in the case of BSSL and equal to (1 – α) in the case of AFX.
